# Hybrid flexible (HyFlex) teaching and learning: climbing the mountain of implementation challenges for synchronous online and face-to-face seminars during a pandemic

**DOI:** 10.1007/s10984-022-09408-y

**Published:** 2022-04-05

**Authors:** Michael Detyna, Rodrigo Sanchez-Pizani, Vincent Giampietro, Eleanor J. Dommett, Kyle Dyer

**Affiliations:** 1 Centre for Technology Enhanced Learning, King’s College, London, SE1 9NH UK; 2 King’s College, London AV, Strand, WC2R 2LS UK; 3 Department of Neuroimaging, Institute of Psychiatry, Psychology & Neuroscience, King’s College, London, UK; 4 Department of Psychology, Institute of Psychiatry, Psychology & Neuroscience, King’s College, London, UK; 5 Department of Addictions, Institute of Psychiatry, Psychology & Neuroscience, King’s College, London, UK; 6 Curriculum & Digital Innovation, Institute of Psychiatry, Psychology & Neuroscience, King’s College, London, UK; 7 School of the Built Environment and Architecture, London South Bank University, London, UK

**Keywords:** Dual mode, Higher education, Hybrid flexible, HyFlex, Innovation, Learning space

## Abstract

In 2020, King’s College London introduced HyFlex teaching as a means to supplement online and face-to-face teaching and to respond to Covid-19 restrictions. This enabled teaching to a mixed cohort of students (both online and on campus). This article provides an outline of how such an approach was conceptualized and implemented in a higher-education institution during an intense three-month period over that summer and prior to the limited re-opening of the university campus. This was a new approach that offers a number of pointers for reflection and provides key insights in on this novel learning environment and the physical and pedagogical contexts in which learning can occur. Technical implementation factors are detailed, along with both reflections on challenges and solutions. Pedagogical issues such as cognitive load, social presence, and resolving the issues of a cohort spread across two locations are discussed. While we should be mindful of the limitations of this relatively-specific research, and shouldn’t therefore over-extrapolate our findings, one key finding is that delivering Hyflex is associated with a higher cognitive load. Further, the audio quality of our implementation enhanced the feeling of presence in the learning environment. We recommend providing appropriate technical and pedagogical training, as well as audio-visual and digital education support.

## Introduction

Covid-19 has impacted higher education worldwide (Daniel, 2020), causing a rapid shift from face-to-face to online teaching and assessment and upskilling of staff and students (Sun et al., 2020). While digital education is a priority across the sector, the pandemic required adoption of novel approaches (Lockee, 2021). To avoid this becoming a case of technology dictating pedagogy or seeking impossible requirements, an iterative process was developed.

Several delivery modes were considered across the university. Synchronous delivery fully face-to-face was impractical because of Covid lockdown and social-distancing measures. Synchronous fully online teaching was also considered for lectures, but it was felt that asynchronous lectures were preferable (Daniel, 2020). This was particularly important given that our large number of international students were in multiple time zones. For seminars and workshops, however, as described by An and Oliver (2021); there are complex and dynamic interactions and ongoing changes between people, technology and education; thus meaning the challenge was more complex due to the increased interaction between participants.

Whilst lectures could be delivered asynchronously, seminars needed to happen synchronously. Two options were considered, in addition to fully online. First, a mixed approach involved face-to-face sessions and separate online synchronous sessions, so that students were in groups that were fully online for periods of time but fully face-to-face at other times, and the two modes did not learn together. Second, a hybrid flexible approach allowed students to attend the class on campus or online and both groups learn together synchronously. This approach also is known as HyFlex (Beatty, 2019). We opted to use both approaches in different faculties.

The present article describes how we adopted a multidisciplinary dialogue approach to deliver creative solutions under a series of external constraints by detailing the introduction of HyFlex teaching in response to the pandemic and the creation of a novel learning space.

## Literature review

There is an emerging body of literature about this approach in the UK because of its novelty here in this country, but there are international examples that were conducted before the pandemic. Triyason et al. (2020) outlined design possibilities and challenges of Hyflex whilst Wright (2015) argued that, for HyFlex to be successfully executed, four main factors need to be considered: equivalency (of experiences), reusability, accessibility, and learner choice (of participation mode).

Malczyk and Mollenkopf (2019) argued that students respond and engage well in HyFlex courses, which proved not to be a barrier to social development among students because they had made their own social networks outside lectures. This partially contradicts Koskinen’s (2018) observations that there are barriers to any form of online learning, including being potentially less engaging and being more of a disconnected, passive experience. Furthermore, simply reusing an existing curriculum might not be most effective when being used for HyFlex and might not unite physical and virtual students well enough; but active learning techniques can help with this. Kohnke and Moorhouse (2021) found that students appreciated the flexibility of a HyFlex learning environment but perceived an increase in workload.

When Liu and Rodriguez (2019) evaluated the impact that a similar approach had on students, they found some evidence for the desire of flexibility that this provides. Care must be taken not to over extrapolate these results because their definition of HyFlex did not use synchronous teaching, but rather the flexibility of choice between an in-room synchronous session or an online asynchronous one. This was in line with findings by Abdelmalak and Parra (2016).

The largest review on the subject by Raes et al. (2019) provided an overview of the benefits, challenges and design principles of synchronous Hyflex learning based on 47 research studies. Raes argued that, compared to fully online or fully onsite, Hyflex is a more-flexible and more-engaging learning space. The themes that they identified, including overcoming technical challenges and a need for new pedagogical approaches, were echoed by others such as Zydney et al. (2019). While there are both benefits and challenges with the HyFlex approach, it could allow staff to lecture in rooms with which they are familiar and allow students to have the option of being in the room or access it remotely. A report by Maxwell (2021) showed some ways to mitigate challenges such as lecturer cognitive load, including by using two teachers.

It is important to consider the physical space and its effects on teaching with respect to HyFlex. Leijon and Lundgren (2019) conceptualised different types of space in the HyFlex model, including both physical space and interactional space. The way in which these spaces are designed or adapted is critical for optimal communication, interaction and, as a result, learning. A teacher implementing the HyFlex model needs to be able to communicate whilst interacting with all the different spaces. The teacher’s movement within the space and the variety of teaching styles must be considered when implementing a design and fitting additional equipment. Critically, as Binnewies and Wang (2019) note, students often appreciate the flexibility that HyFlex provides, but this is constrained by the available technology. Prior studies (e.g. Butler et al., 2017) have shown that audio quality (in the sense of not having too much noise) is important in determining the quality of a learning space. Mantooth et al. (2021) reinforce the point that, for effective learning environments, any novel technology needs to be paired with appropriate pedagogy, which is a point that this research endorses. There is an emerging literature on student perceptions of this form of learning environment, with Keiper et al (2021) identifying examples of positive feedback with a given tool in a specific context.

One obvious question is what the existing literature states about how to create a HyFlex environment and whether the design deviates from current in-room teaching considerations. Zydney et al. (2019) gave an overview of their implementation process, and Bower et al. (2017) describe a pilot study in an Australian university, which revealed that using a single screen provided some measure of a sense of ‘presence’ for both groups of students.

## Implementing HyFlex: considerations

### Deployment

**Fig. 1:**
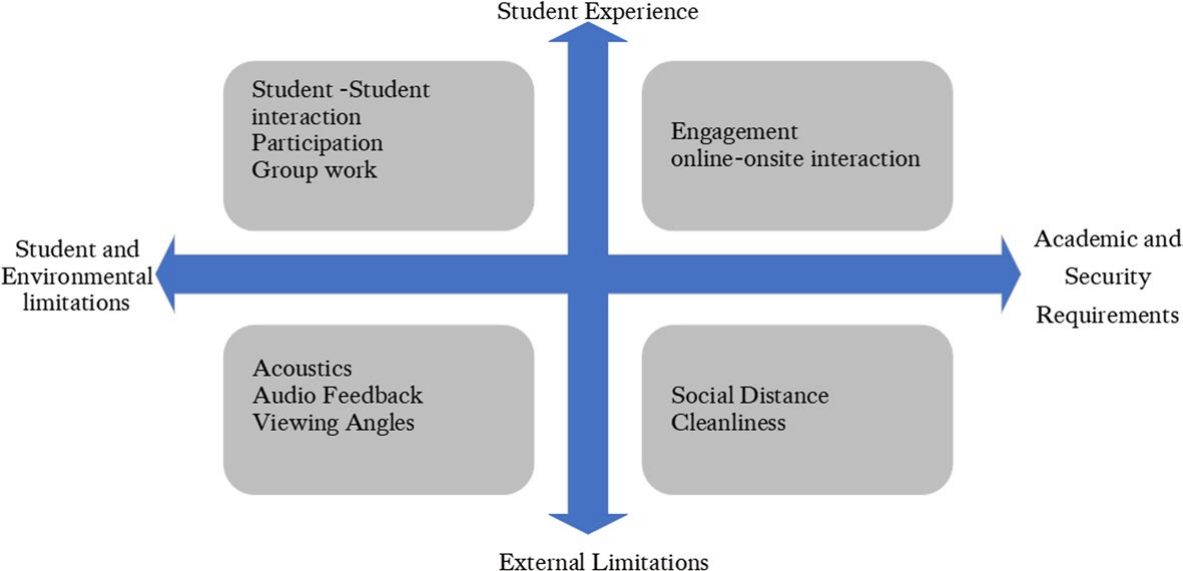
Competing requirements and challenges in implementing HyFlex

It was proposed that implementing the HyFlex model should follow a holistic approach. For example, Flavin (2020) stated that, in the context of virtual learning environments, simply adapting technology to current practices is not disruptive and maintains the status quo; therefore, change must come from pedagogical practices. This is a sentiment echoed by Gogia (2020), who discussed the risks of making HyFlex all about investing in the technology and forgetting about the pedagogy. It was agreed that, in order to produce an efficient solution, a multidisciplinary decision-making process was necessary. Shang (2005) identified four stages in the knowledge acquisition process (namely, planning, extraction, analysis and verification), with this process being transferable from technology design to other domains as described and extended by Guenther (2013). There were also competing requirements and challenges of implementing HyFlex, shown in Fig. 1.

**Fig. 2:**
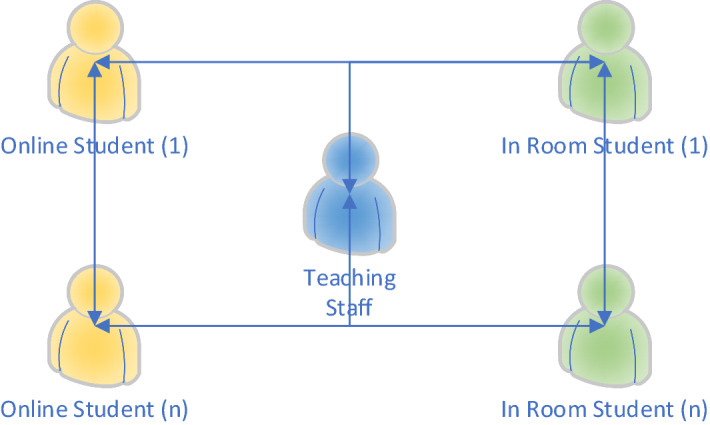
Synchronous interactions required by HyFlex

The approach was informed by the pedagogy-space-technology framework from Radcliffe (2009) because each area is important and affects and informs the others. Finding the right balance between pedagogy, technology and compliance with current regulations resulted in competing requirements and limitations. These were between the user experience and constraints from safety, practicality, budget, quality, the room (acoustics and lighting) and audiovisual technology as summarized in Fig. 2.

Because Kings’ College London wanted to extend the number of spaces as much as possible to increase the number of classes, the solution had to be relatively inexpensive and easy to deploy in order to have it ready by the start of the academic year. It is important also to consider the two-metres social-distancing requirement.

### User experience

**Fig. 3:**
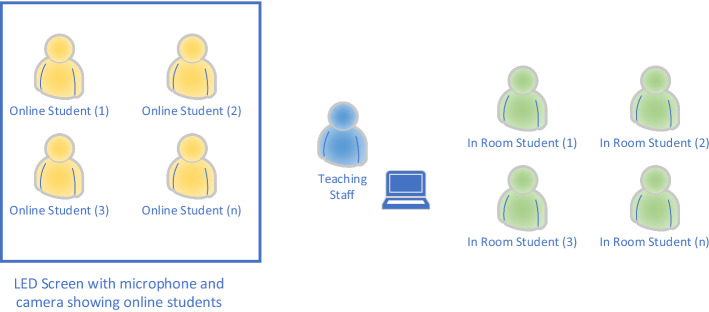
Simplified outline diagram of the HyFlex system

The requirement of using the spaces for seminars (i.e. places to encourage debate as well as student–student and teacher–student interaction) was in direct contradiction with the audio and video quality requirements. These are limited by room characteristics and available technology, and the flow of the interaction is best appreciated in Figs. 2 and 3.

Other issues that were considered were the notion of social presence (as discussed by Szeto & Cheng, 2016). Students attending on campus could potentially have more non-verbal modes of communication than online students. The use of video screens was aimed at reducing these discrepancies. Other issues to consider were the use of the technology itself because, to be confident, educators need to test and practice, making the technology they intend to use work for the teaching strategies that they want to enact, and they need to plan for contingencies if anything goes wrong.

The lecturer is connected to the AV system and all participants can be both seen and heard.

### Audio and video

The basic summary of synchronous interactions required by HyFlex is outlined in Fig. 2, and a simplified outline diagram of HyFlex is shown in Fig. 3. With regards to the in-room experience, George and Youssef (2012) showed that audio quality is important in a learning space. Yang et al. (2013) studied how higher-education classrooms impact student satisfaction and performance, showing acoustics to be the most-important factor. An investigation by Peng et al. (2015) revealed a strong relationship between the acoustics of the room and intelligibility. The above is consistent with research in electroacoustics and subjective acoustics, such as that discussed by Alves et al. (2010) who examined a method to improve intelligibility in noise and objective metrics such as the Speech Transmission Index (STI), and who used as a method to evaluate the degradation of the acoustic channel developed by Steeneken and Houtgast (1980). Morales (2014) validated these results in real conditions and Morales et al. (2018) proposed a reviewed spectrum of this index. Fazenda et al. (2015) investigated a blind methodology to evaluate the sound quality of audio quality. Our implementation aimed to ensure that high-quality audio allowed distanced learners to participate fully in the pedagogical experience as if physically present in the learning environment.

One particular concern for this HyFlex project was the use of face masks and their potential effects on quality of the audio captured, although Mendel et al. (2008) could not find their hypothesized degradation on speech perception.

Previous studies of HyFlex-like environments have not necessarily focused on video quality, but on how the image should appear, and discussed camera position and the importance of not limiting the academic’s movement (Zydney et al., 2019). These are all in accordance with the requirements of Kings College London’s teaching staff and students based on feedback received (Dommett et al., 2019). This creates some additional pressures by adding a new dimension (movement), which has a significant implication on the audio capture requirements; the larger the distance between the source and the receiver (academic-microphone pair), the lower the sound (pressure). The use of a higher definition camera presents further challenges.^1^

### Video conferencing technology

Zoom,^2^ Echo360,^3^ and MS Teams^4^ were all considered as possible digital tools to facilitate HyFlex. There were some security and licensing questions around Zoom at the time, while Echo360 was not properly set up for live streaming at King’s. MS Teams had some limitations regarding interaction and group work, with Breakout Rooms not being initially available (although this was later resolved). Adobe Connect^5^had a reasonable track record as a fully-virtual classroom, but the interface and licensing pushed it out of the selection. We eventually settled on MS Teams. We also recommended that in-room students have their own devices so that student faces could be seen clearly.

### External limitations (COVID-19 specific)

The pandemic created an additional set of challenges that were external to the design and mostly focused on safety. The UK government established guidance for HE (www.gov.uk, n.d.) which included various safety measures that were followed in full.

Summary of key design points for implementation: audio and video.

- Good-quality and intelligible sound for and from every online student, for and from every student in the classroom, wherever they sit, and to and from the academic.
- Classroom should be visible to online students, who require a (virtual) presence in the room that is available to at least the academic and potentially to all.
- Security: Equipment needs to be safe to use and online interaction needs to be restricted to the students in that class.
- Portability, budget and scalability: Systems need to be simple and movable, capable of integrating with the technology available in the rooms, and expandable to as many rooms as possible.
- Hygiene (Covid-19 specific): Two-metre distance between students and wearing face masks were required.
- Surface touching had to be minimized, with any surface being thoroughly cleaned before the next use.
- Interactions: Group interaction and discussion between all the students and academics should be possible (see Fig. 3)
- Graphics (pictures, slides, tables, etc.) and text should be visible for online and on-site students.
- Lecture with academic in room should be available to students online and on-site
- Lecture with academic online should be available to students online and on-site.

## Analysing how to implement HyFlex

Before moving forward with any implementation, some basic decisions (D), recommendations (R) and assumptions (A) needed to be made:

- Every student must have access to a portable device that allows interaction (R-A).
- MS Teams was eventually decided upon, mainly because of the institutional inertia and because it was the main web conferencing tool available (D).
- The number of cleaning staff could need to be increased to meet demand (R).
- Appropriate space, cleaning and timetabling was needed to ensure space, social distancing and cleanliness (R).
- The university needs to consider how to deal with the cognitive load associated with an online and face-to-face session simultaneously, for both staff and students (R)
- The university needs to consider how to ensure equity for all students and parity of experience when online and face-to-face simultaneously, so that both groups of students get the same quality of education (R)

### Technical set-up

All the factors above were considered in devising the outline of an audiovisual approach. The integration with the current audiovisual systems in the rooms was planned to allow for staff members to show a presentation on the large screen already present in the room. Using a movable secondary large screen and a laptop with extended desktop allowed online students to be shown to the lecturer and their peers in the room. To cover the room and be able to fulfill the requirements of the video input, a 4 K camera equipped with an artificial intelligence engine, that provides auto focus and pan/tilt framing of the loudspeaker, was added to the top of the screen. We used a specially-designed mount to allow mechanical adjustments and the camera was integrated with the laptop in the room. Several options for audio were considered as shown in Table 1.

**Table 1:** Deciding the type of microphones

Set-up	Pros	Cons
Wireless hand-held microphones	Good audio given the proximity to the source	Difficult to maintain, storage, hygiene concerns, talker needs to hold the microphone
Wireless clip-on microphones (tie or lavalier microphones)	Good audio, user keeps hands free	Difficult to maintain, storage, hygiene concerns, prone to audio feedback
Push to talk microphones	Good audio, increases control as the chairperson can mute participants	Hygiene

Two approaches were considered. The first involved acoustic room treatment to minimize reverberation and noise. This alternative was thought to have two main problems: too much absorption would have an impact of the sound pressure, and people seated at the back of the room might struggle to hear others in the room (Hodgson, 1999). As a result, this would improve the experience for students online at the detriment of those on site. A second option was to use beam-forming microphones, but these would be difficult to use in rooms with high reverberation time. Therefore, the chosen approach was to combine beam-forming microphones and rooms perceived to have low reverberation.

Finally, all systems were integrated with the existing room AV equipment and used through a single USB-C presented to a central desk with a standard Kings’ College London laptop.

**Fig. 4:**
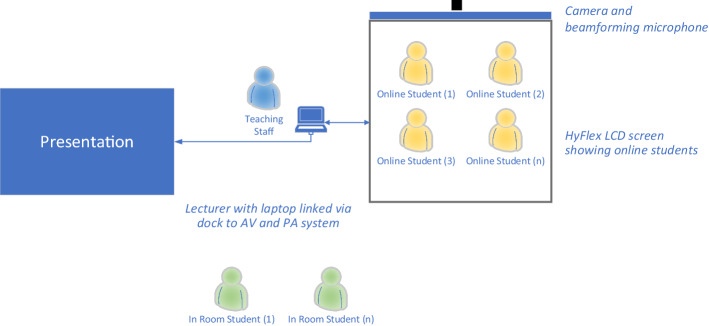
Outline schematic

In the outline shown in Fig. 4, audio from students in the room is captured by the beam-forming microphones and video via the 4 K camera system. The lecturer can be seen either through the laptop camera or the camera on the HyFlex screen. Students in the room can see online students via the large LCD screen and the presentation on the main screen, and they can hear content and online students via the built-in sound system. The lecturer’s laptop connects via a dock to the existing AV system. All of the sound and audio from the HyFlex system is integrated into the existing system. Online students are connected via MS Teams and can see and hear all participants*.* What is novel about this approach is the high resolution 4 k camera, the second screen, and the beam-forming microphones which ensure good capture of in-room students.

**Fig. 5:**
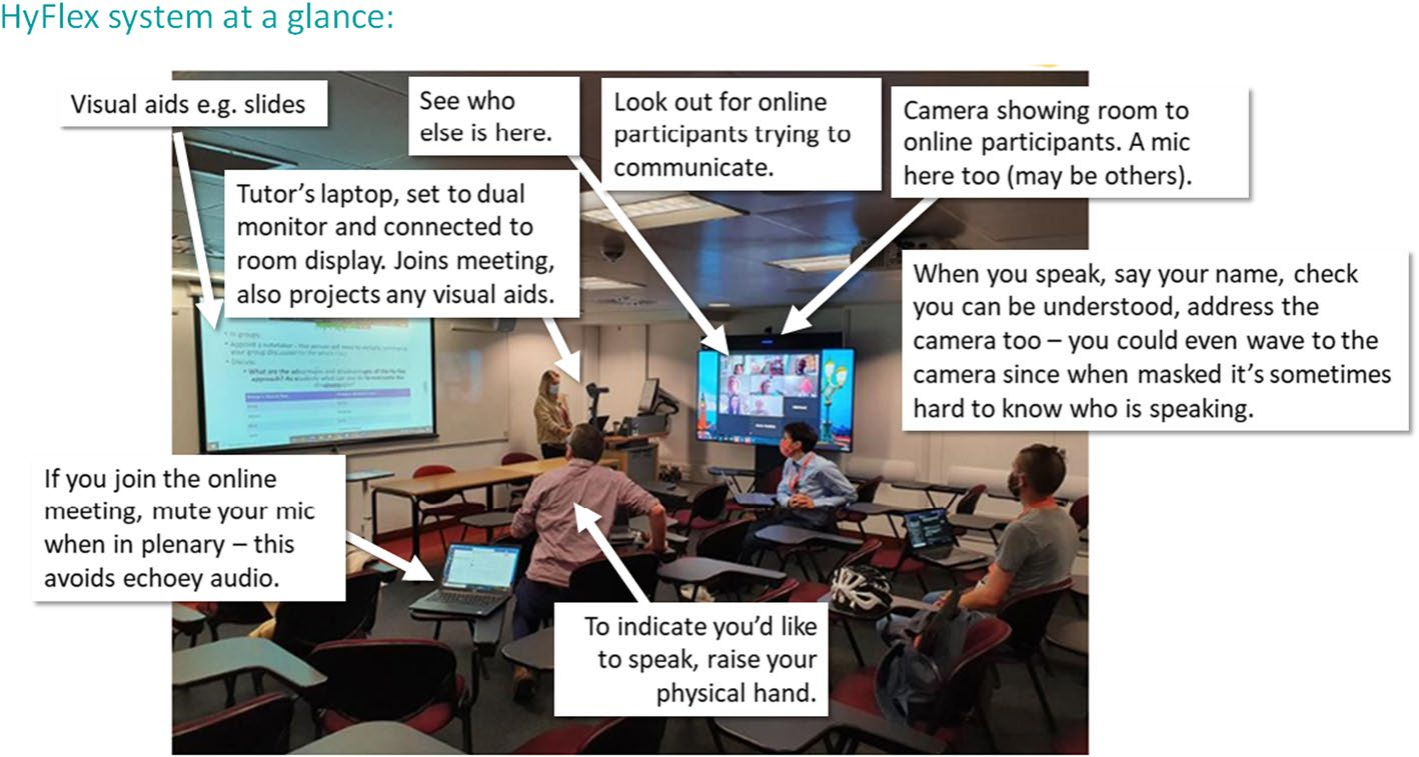
Outline of the Kings’ College London HyFlex system from guidance material. (Image credit: Mira Vogel)

Figure 5 shows the view from the student perspective, and gives an overview of how the room looks and a general sense of the space. Leijon and Lundgren’s (2019) notions of a shared space between online and on campus-students can be seen here.

## Technical testing

### Methodology

Four mock sessions were held to model and test the simulated learning environment, with academics presenting and professional and academic staff attending as students both onsite and remotely. Presentations included slides, videos, and interactive discussions both from presenters in the room and online.

Initial testing was a pilot undertaken in a large room (126 m^2^) to test prototype equipment and to try out different teaching combinations. After several further tests, we defined the spaces to be used as follows:

1. T1: small rooms (surface < 55 m^2^)
2. T2: medium rooms (55 ≤ surface ≤ 105 m^2^)
3. T3: large rooms (surface > 105 m^2^)

Additional testing was conducted to ensure that the main teaching activities could be conducted with satisfactory outcomes. There were a number of specific teaching activities (e.g. lecturer talking to the group; pair discussion; group discussion; showing a presentation; small groups in breakout rooms) to which academics had previously agreed as standard for a seminar, and these were all tested using the HyFlex set-up. Initial piloting with senior academics and specialist staff led to a decision to restrict to PGT and cohorts of no more than 30 students.

After running the mock sessions, several actions were drafted. Teaching staff were advised to try to reduce noise in the room and, if necessary, to repeat what students said. Another issue identified was the need for more engagement with those in the room. Recommendations to address this included providing icebreaker activities at the start and reflecting on how to bring the group together.

It was also highlighted during the test sessions that it can be a challenge to engage online students, and so it was recommended that a graduate teaching assistant help to manage the chat and act as an assistant although, in the initial stages, there were no resources for this. Because it was also established that allowing lecturers the freedom to bring their own laptop created more problems than it solved (because of software and hardware compatibility), a dedicated standard laptop was provided to ensure simplicity and ease of use. Audio was sufficiently strong in our set up that users commented on the quality was high and increased the sense of immersion in the learning environment for remote users.

### Installation, deployment and further analysis

The system was designed so that it could be deployed in a short period of time and we aimed to install 34 HyFlex Rooms between August and September.

#### Guidance and induction

Over the summer of implementation, a huge effort was made to research, plan, rewrite, and redraft written guidance. This was put together in the form of staff and student sites on the institutional virtual learning environment (VLE). In addition, in-person and online induction sessions were held to provide staff with the space to reflect on best practices, with thoughts from the literature.

There are a number of issues that were raised during technical and teaching testing and some of the recommendations for staff that emerged are recorded in Table 2.

**Table 2:** Recommendation for each staff issue

Staff issue	Recommendation
Not knowing the technology	Staff should have already completed training in MS Teams, and they should physically try everything prior to the session with a member of the AV team in the room
Pedagogy: Students not feeling engaged online and face-to-face	Staff should welcome all students and aim for smooth transitions between sections of their session. Lecturers should ensure that their eyes look equally at the camera and at the students in the room to ensure equity for all students
Lack of clarity for students in the session	Staff should plan the session appropriately and advise students to be patient. Further, it is important for staff to recognize that things might not work perfectly initially
Feeling overwhelmed	Staff should avoid doing too much too quickly, and keep it simple. Further, staff should prepare the session well ahead of time and prepare themselves to experience potential issues and delays. Lecturers can have an AV member of staff available at the first couple of sessions. We recognize that the HyFlex approach would not suit everyone and every teaching session
Safety (Covid-19-related)	All participants should stay two metres apart from each other and wear a face covering. All staff were made aware of Kings’ College London’s guidance on Coronavirus safety and advice on staying safe on campus
Staff wanting to move around the room	Staff should be aware of where the camera is facing and adjust it if appropriate
Students online feeling ignored	Staff should make a point of welcoming students and talking to online students throughout the session

### Pedagogical issues

A total of 104 induction sessions were held with academic staff (32 in 2020 and 72 in 2021) so that various ideas, issues and concerns could be discussed. It is crucial to take heed of Gogia (2020) who noted that pedagogy can often be overlooked.

Looking at the student experience, there are advantages for each group (online and on-campus) at different times and in different situations. Online students can have some advantages when using HyFlex: proximity to the microphone so that everyone online can be heard clearly; and access to other reading materials such as online books and articles. On-campus students can have other benefits such as the ease of familiarity with their peers and greater ease of making face-to-face connections. We should be mindful that (with some exceptions) both groups could swap over time in that online students could choose to come on campus and vice-versa. Getting them to experience ‘both sides’ could allow students to be a good source of feedback for educators and we would certainly encourage a dialogue between students and lecturers as Abbot and Cook-Sather (2020) suggest.

One of the challenges discussed above was ensuring that online students felt part of the group. As Oh et al. (2018) argued, there are a number of factors that impact social presence in an online environment, and one way to ensure presence and connection is for participants to both show their face and use eye contact.

In general, we would suggest a 2 × n matrix of specific local challenges and solutions. Pedagogical challenges/issues can be noted in a collaborative document, and then solutions can be proposed and discussed.

## Discussion

### Interpretations

Overall, it is clear that this new approach to a learning environment requires new technical and pedagogical approaches. This research has provided some important novel findings, particularly on how dual screen set ups can be used for synchronous teaching and learning and how implementation challenges can be overcome in this new learning environment. A hybrid learning environment needs be considered carefully in designing equipment, setting up, and considering how both academics and students engage with such a space.

From inception, it was clear that, while it would indeed be possible to provide synchronous online and face-to-face delivery within the same session, this would not be easy to enable and it would involve technical challenges. The current work is limited to the challenges and deployment that we faced at one institution before HyFlex was first used in teaching and, therefore, is limited in its scope. We should also note that experience and needs will be different because some academics and students might use Hyflex regularly, whilst it could well be a one-off for others. It is not possible to generalize from our experience/results in a pedagogical and technical setting. Further such studies are ongoing at our university and beyond.

HyFlex was set up in the middle of a pandemic and this obviously presented additional challenges (e.g. all relevant government and WHO guidelines changed regularly and were followed). The challenges changed over time, as did the nature of the solutions, in an iterative fashion. We feel that, after the Covid-19 pandemic, there will still be an important use for this approach, albeit on a smaller basis, in situations where students are overseas, to provide the added flexibility for students to study synchronously from home where appropriate, and for working environments of the future. The sense of immersion and audio quality of this specific implementation could help to provide a fully-shared learning context for overseas learners.

### Implications

Ensuring equity for all students is important and providing an experience that all students feel is beneficial and fair to them is crucial. If students attending face-to-face feel that they are getting a better experience than those attending online or vice versa, this could be detrimental to student equity. The written guidance that we produced focused on ways and mechanisms to address this, including simple reminders for staff to ensure equity to both groups, being mindful of eye placement (Oh et al., 2018), and ensuring that only one person speaks in the room at a time so that there are fewer noise issues. In general, in looking at student equity issues, we adopted the methodology suggested by Sellars (2017) of asking staff members to reflect on potential solutions to address this issue. Binnewies and Wang (2019) have written eloquently on the issue of student equity using HyFlex, and our research builds on their approach, which promotes active learning techniques. Ways of embedding equity could be to ensure that both online and on campus students raise hands (real or virtual) before speaking, and that in-room students also have a device through which to engage with online students.

Perceived Ease of Use (PEU) is a key metric in whether technology can be accepted by staff and students and provide educational benefit (Venkatesh & Davis, 2000). If the system is too convoluted or complicated, this will detract from the educational benefit (Cilliers & Pylman, 2020). Concerns over complexity should not be overlooked, and we should aspire for a system that is widely accepted as simple and straightforward to use. Further evaluation of PEU with HyFlex is highly recommended to determine if the setup requires to be simplified further.

### Limitations

We should be mindful of the limitations of this research. We shouldn’t over-extrapolate our findings and remain mindful that their primary relevance is to a specific time and place. Because our experience might not be generalizable to other institutional contexts, understanding the specificity of this research is important. We should also recognize the time constraints under which the work was carried out. In general, it is important to keep in mind the limitations of any HyFlex implementation (Raes et al., 2019), and not transform this approach into a marketing tool by simply listing positive-sounding adjectives (e.g. flexibility, interactivity) without also recognitising its limitations, both technical and pedagogical.

### Recommendations

One key point found throughout our testing and in the literature is that delivering Hyflex seminars is associated with a high cognitive load; lecturers must consider the points that they are making, the overall structure of the session, the audience, what and how they want to say, being inclusive, etc. In going forward, we recommend providing appropriate technical and pedagogical training, as well as audiovisual and digital education support.

We also feel that further research is needed and would suggest that further testing is conducted. At Kings’ College London, four selected rooms (3 T1 and 1 T2) will be fully tested acoustically, impulse responses will be calculated from the position of the microphones, and a survey will be used to collect opinions from staff and students and to explore links between the subjective data and the objective parameters of the room. Future evaluations could use the framework of Hao et al. (2021) to quantify effects on the space as a learning environment, (distinct from pedagogical approaches). We also intend to undertake a full technical evaluation of the audio quality following on from comments during the implementation concerning how this increases the sense of immersion in the learning environment.

## Conclusion

In conclusion, it is clear that the Covid-19 pandemic has produced a push for developing and evaluating new approaches to the university classroom. The HyFlex approach was completely novel to Kings’ College London and largely novel to the UK. We identified a number of challenges, particularly around technology, student equity, acoustics, and pedagogy. Further evaluation is needed into how effective this was as an approach, especially in the context of real teaching sessions and outside of the Covid-19 context.

## Footnotes

1 Higher-definition cameras require higher demand for bandwidth. ITU (2018, p. 6) recommends 100 Mbits/s for an SD signal and 400 Mbit/s for HD and 1 Gbit/s for 4 K. Similar higher resolutions require larger displays (AVIXA and ITU).

2 https://zoom.us/.

3 https://echo360.com/.

4 https://www.microsoft.com/en-gb/microsoft-teams/group-chat-software.

5 https://www.adobe.com/uk/products/adobeconnect.
